# The compensation for nonlinear friction of DDVC flange-type rotary vane steering gear

**DOI:** 10.1371/journal.pone.0207018

**Published:** 2018-11-12

**Authors:** Lihua Liang, Luyang Wang, Jingfu Wang

**Affiliations:** College of Automation, Harbin Engineering University, Harbin, Heilong Jiang, China; Stellenbosch University, SOUTH AFRICA

## Abstract

This study reports on the direct drive volume control flange-type rotary vane steering gear (DDVC-FRVSG), a promising component with superior advantages of compact structure, powerful vibration absorption, and simple control for application in the controlling course and posture of a vessel. The ability of the DDVC-FRVSG to satisfy the accuracy requirement of the vessel is limited by nonlinear friction. This study proposes two compensation methods to compensate for the nonlinear friction. We establish the mathematical model and the transfer function of the steering gear system and the mathematical model of nonlinear friction on the DDVC-FRVSG system based on the principle of the DDVC-FRVSG. A high-gain proportional–integral–derivative control strategy and another method using the self-adaption robust control strategy is proposed and studied both theoretically and experimentally to suppress the nonlinear friction. With the “no-compensation state” as a benchmark, our measured results by prototype testing has proved that both methods can compensate for the nonlinear friction, with the second method showing a better performance of up to 78.85% increase compared to that of the 41.65% shown by the first one. The outcome of this research will contribute to the rapidity and stability of the DDVC-FRVSG.

## Introduction

In addition to the traditional function of course control, the ship rudder makes increasing demands on the rudder roll stabilization in its own scope of application [1], aiming to steadily, rapidly, and accurately track the rudder angle commands [2]. As mentioned in the references, the rotary speed of the rudder must reach 15°/s for rudder roll stabilization [1]. The performance of control on the rudder angle position directly affects the accuracy and robustness of the ship’s course control. The performance of the rudder roll stabilization affects the ship’s sailing stability [1]. The increasing demands on the rotary response speed and ship stability have established a higher requirement for dynamic performance, such as rudder response speed and frequency response. [1]. Therefore, the rudder must be maintained with excellent control quality [3].

**Fig 1:**
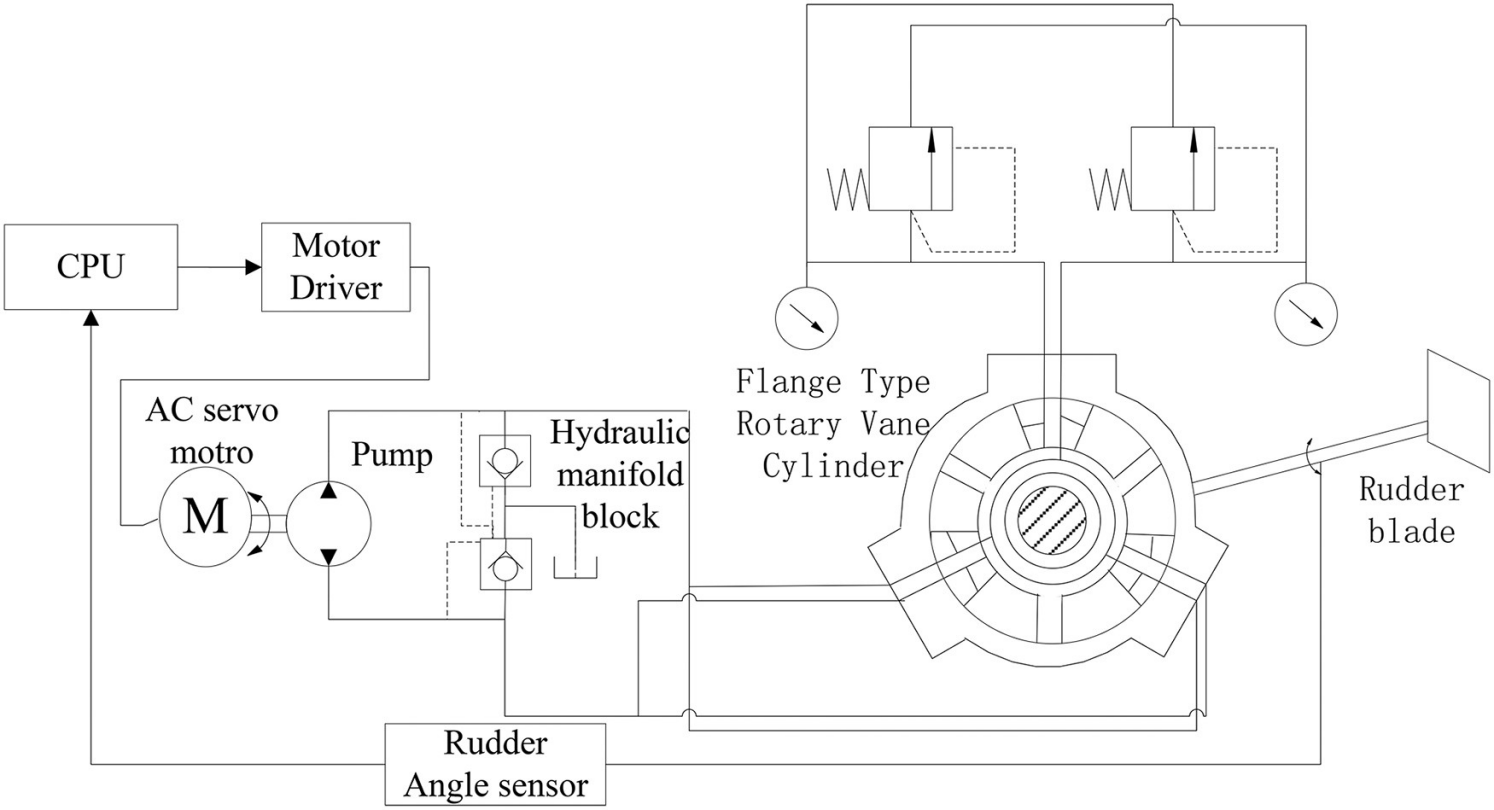
Schematic of the DDVC–FRVSG.

As a result of its structural characteristics, the rotary vane steering gear with a flange-type structure has a better sealing performance than that with an end shield-type structure [4, 5]. Moreover, it also has a very prominent absorbing performance against various types of vibrations and shocks under water [5]. Therefore, the direct drive structure of the rotary vane rudder cannot only integrate the flexibility of servo motor control and the high efficiency of hydraulic volume control, but also greatly simplify the system structure in principle, as prominently featured in its simple structure, compact size, high system efficiency, low power consumption, low vibration noise level, high reliability, etc [6]. The direct drive volume control flange-type rotary vane steering gear (DDVC-FRVSG) combining the “DDVC system” with a “rotary rudder of flange-type structure” is a type of electro-hydraulic servo steering gear that integrates all the advantages of the DDVC drive mechanism and the rotary vane rudder of the flange-type structure [5]. Its dynamic performance must be improved to maintain the DDVC-FRVSG with the excellent control quality necessary for a rotary vane rudder [5]. Fig 1 shows a schematic of the DDVC-FRVSG system.

The nonlinearity of the DDVC-FRVSG system mainly derives from its operating environment (i.e., the nonlinearity of the external load moments applied on the rudder) [7–9]. The external load moments applied on the rudder generally consist of the hydrodynamic moment, the friction torque, the inertia moment, and other external load moments [10, 11]. Among which, the friction torque is the essential nonlinear factor affecting the dynamic performance of the servo system [12]. The clearance and the dry friction in the servo system make the nonlinearity of the rudder structure system more obvious; if serious, the nonlinearity may even cause frequency response characteristics beyond the design index or the self-excited vibration and consequently reduce the rudder system’s operating efficiency and quality [13]. The presence of friction makes it difficult for the rudder to rapidly start up and track the designated position, as scheduled in the design requirement [13]. Moreover, it creates the “dead zone” and “stick-slip” phenomena in the rudder response process, resulting in the rudder’s motion stability, course stability, and automatic steering sensitivity failure to meet the related design requirements. Friction is a common nonlinear phenomenon; hence, it is inevitable in the relative motion process for almost every type of mechanical system [14]. In the DDVC-FRVSG system, friction generally occurs on the rudder bearing between the rudder shaft and the rudder body [15, 16]. Friction will generally reduce the system’s dynamic performance and create several deviations from expectations, such as the limit cycle, the dead zone, and the flat-topped curve [17–19]. Hence, in-depth research studies on the friction action process in the rudder and its compensation method are necessary for the DDVC-FRVSG system [20].

Before the friction force may be properly compensated in a suitably selected manner, its variation characteristics and effects on the system should be analyzed in detail [21]. In the literature [22–33], the LuGre model provides the most complete information, and can accurately describe the static and dynamic performance of friction. Hence, the compensation method is commonly based on the LuGre model [34–38].

Considering that friction exists in the relative motion process for almost every mechanical system [14], many methods for friction compensation have been attempted in different types of servo control systems [30]. De WCC. proposed the Bristle model to identify the parameters of the motor servo control system, analyzed the influence of the load type change on the friction model through experimental studies, and designed an adaptive regulating controller for this model [26]. Kebairi A proposed friction modeling and identification methods for a Bosch GPA-S electro-mechanical actuator [30]. Begin-Drolet designed a robust control approach for periodic systems. Wang CW proposed a practical nonlinear robust control approach of an electro-hydraulic load simulator. Xu L proposed an adaptive robust control method to compensate for the nonlinear friction during the operation of a mechanical servo system that obtained ideal compensation effects. [39–45].

Given that the electro-mechanical servo system and the DDVC system have many similarities in their operating processes, they may have many common problems. Therefore, this study considers the possibility of applying the friction compensation approach that is effective for the electro-mechanical servo system to the DDVC-FRVSG driven by the DDVC system.

First, this study introduces the system composition of the DDVC-FRVSG and sets up a mathematical model to establish a mathematical model that can both describe the system’s true dynamic process, including the nonlinear friction process, and apply control methods to successfully obtain the control effect. Next, the operating principle is summarized. A LuGre-based friction model is set up for the friction moment interference with the DDVC-FRVSG to explore a high-gain proportional–integral–derivative (PID) compensation strategy for the friction moment on the DDVC-FRVSG system. Finally, a self-adaption robust control strategy of friction compensation is established for the friction moment on the DDVC-FRVSG system. The compensation effects from various types of compensation strategies are studied to determine an effective means to eliminate the interference of low-speed nonlinear friction with the DDVC-FRVSG. Regardless of the hydrodynamic action, this study describes the system’s dynamic performance as a test curve of the rudder angle response. All results are verified through experiments.

## Materials and methods

### System description

**Fig 2:**
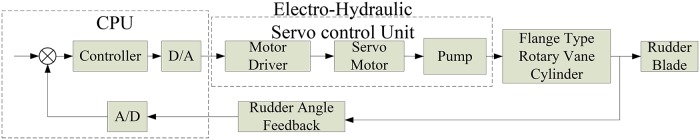
Schematic block diagram of the DDVC–FRVSG.

Fig 2 presents a block diagram of the system, where three parts can be discerned. The computer control part includes a host computer, a quanser, and a rudder instruction feedback device. The electro-hydraulic part consists of an alternating current (AC) servomotor and driver, a fixed displacement pump, a hydraulic manifold block, an oil tank, and hydraulic pipes to connect all the hydraulic components. The flange-type rotary vane steering gear part contains a flange-type rotary vane cylinder, the rudder, and other devices.

The fix displacement pump and the flange-type rotary vane steering gear are connected by hydraulic pipes, forming a closed hydraulic circuit. At the other end of the pump, an AC servo-motor drives the pump rotation through the instruction from the controller to supply oil to the flange-type rotary vane steering gear, thereby driving the rudder rotation. The rotary speed of the rudder is controlled by the AC servomotor, and the direction is turned via the motor shaft rotation. Therefore, an angle sensor is used in a closed-loop feedback to achieve the purpose of controlling the steering gear rotation.

### System modeling

This section addresses the system modeling in Fig 2. The system input is the order rotary speed of the servomotor, and its output is the shaft angle of the rudder. The objective is to design a model that accurately describes the relationship between the order rotary speed of the servomotor and the output of the shaft angle. The following assumptions should be introduced before the design process:

1. The mass effect of a vibrating flow pipe, the pressure loss, and the dynamic effect are ignored.
2. The total volume (including pipes) of the input oil chamber is equal to the output oil chamber; the pressure around the chamber is constant; and the oil temperature should not rise. The variation in the volumetric modulus of elasticity is ignored.
3. The effect of structural flexibility and the pressure loss of hydraulic components are ignored.

The model of the rotary vane may be described using the fluid continuity equations below.

In the DDVC-FRVSG system, *Q*_*L*_ is from the rotation of the pump (i.e., the rotary of the servo motor). The model of the rotary vane may be described in Eq (1) according to the fluid continuity equations and the torque–equilibrium equations.

$$\begin{array}{c} Q_{L}=nD_{p}=\overset{•}{\theta_{m}}D_{p} \end{array}$$ (1)

$$\begin{array}{ccc} nD_{p} & - & C_{ip}\left(p_{1}-p_{2}\right)-C_{ep}p_{1}-C_{im}\left(p_{1}-p_{2}\right)-C_{em}p_{1}\\ & = & D_{m}\frac{d\theta }{dt}+\frac{V_{t}}{4\beta_{e}}\frac{dp_{1}}{dt}+(C_{im}+\frac{1}{2}C_{em})p_{1} \end{array}$$ (2)

The torque–equilibrium equation between the rotary vane and the external load can be given as follows:

$$\begin{array}{ccc} T_{g} & = & \left(p_{1}-p_{2}\right)D_{m}=J_{m}\frac{d^{2}\theta }{dt^{2}}+B_{m}\frac{d\theta }{dt}+G\theta +T_{friction}+T_{L} \end{array}$$ (3)

This is true even if the servomotor is a standard direct current (DC) motor, which may be described by the following linear equations:

$$\begin{array}{c} U=k_{ac}\left(R_{a}I+L\overset{•}{I}+k_{e}\theta_{m}\right) \end{array}$$ (4)

$$\begin{array}{c} T_{n}=k_{m}I=J_{T}\overset{••}{\underset{m}{\theta }}+D\overset{•}{\theta_{m}}+T_{load} \end{array}$$ (5)

Thus, according to Eqs (1)–(5), the abovementioned assumptions, and the Laplace transform, the transfer function can be described by Eq (6).

$$\begin{array}{ccc} G_{p}\left(s\right) & = & \frac{\theta (s)}{N(s)}=\frac{4\beta_{e}D_{m}D_{p}}{V_{t}J_{m}s^{3}+V_{t}B_{m}s^{2}+4\beta_{e}D_{m}^{2}s}\\ & = & \frac{4\beta_{e}D_{m}D_{p}}{s(V_{t}J_{m}s^{2}+V_{t}B_{m}s+4\beta_{e}D_{m}^{2})}=\frac{\frac{D_{p}}{D_{m}}}{s(\frac{s^{2}}{\omega_{h}^{2}}+\frac{2\xi_{h}}{\omega_{h}}s+1)} \end{array}$$ (6)

### Nomenclature

Table 1 lists the nomenclature for this paper.

**Table 1:** Nomenclature.

Symbol	Significance	Units
*D*_*p*_	Displacement of pump	*m*^3^/*rev*
*n*	Rotary speed of pump	*rev*/*s*
*D*_*m*_	Radian displacement	*m*^3^/*rad*
*β*_*e*_	Volumetric modulus of elasticity	*Pa*
*θ*	Angle of rudder	*rad*
*V*_*p*_	Volume of high pressure chamber of pump	*m*^3^
*V*_*g*_	Volume of pipes	*m*^3^
*C*_*i*_*p*	pump coefficient of internal leakage	*m*^3^/*Pa*
*C*_*e*_*p*	pump coefficient of external leak	*m*^3^/*Pa*
*C*_*i*_*m*	rotary vane cylinder coefficient of internal leakage	*m*^3^/*Pa*
*C*_*e*_*m*	rotary vane cylinder coefficient of external leakage	*m*^3^/*Pa*
*p*_1_	Pressure of high pressure chamber	*Pa*
*p*_2_	Pressure of low pressure chamber	*Pa*
*T*_*g*_	Torque of rotary vane steering gear	*N*⋅*m*
*J*_*m*_	Totality rotary inertia residual to the cylinder and load	*kg*⋅*m*^2^
*B*_*m*_	Viscous damping coefficient	*N*⋅*m*/(*rad*/*s*)
*G*	Loading spring stiffness	*N*⋅*m*/*rad*
*T*_*friction*_	Friction torque of steering gear	*N*⋅*m*
*T*_*L*_	External torque beside friction	*N*⋅*m*
*D*	Damping coefficient of motor shaft	*N*⋅*m*/(*rad*/*s*)
*J*_*T*_	Rotary inertia residual motor shaft	*kg*⋅*m*^2^
*T*_*n*_	Electromagnetic torque of motor	*N*⋅*m*
*θ*_*m*_	Output location of motor shaft	*rad*
*U*	Control voltage of motor driver	*V*
*k*_*ac*_	Proportionality coefficient of control voltage	zero dimension
*R*_*a*_	impedance of motor system	Ω
*L*	Inductive reactance of motor system	*L*
*I*	Stator current of motor	*A*
*T*_*load*_	Loading torque of motor	*N*⋅*m*
*ω*_*h*_	Inherent frequency of system	rad/s
*ξ*_*h*_	Damping ratio of system	zero dimension
*σ*_0*n*_	Bristle rigidity coefficient	*Nm*/*s*
*σ*_1*n*_	Bristle damping coefficient	*N*/*m*
*T*_*cm*_	Coulomb friction torque	*Nm*
*T*_*sm*_	Static friction torque	*Nm*
*F*_*cn*_	Coulomb friction force	*N*
*F*_*sn*_	Static friction force	*N*
*B*_*θn*_	Viscous friction coefficient	*Nm*/*s*
*R*	The radius of vane	*m*
*r*	The radius of each point on the surface of up and down	*m*
*R*_*r*_	The radius of rudder shaft	*m*
*rθ*_*s*_, *Rθ*_*s*_, *R*_*r*_ *θ*_*s*_	Stribeck speed	*rad*/*s*

## Friction and its effect on the system

In the DDVC-FRVSG system, friction includes: 1) friction in the system structure when the system is under no load condition, 2) friction for the actuator when the system is under no load condition, 3) friction caused by the load, and 4) friction caused by external disturbance. Eq (7) describes the friction torch in the DDVC-FRVSG system. These frictions cause the phenomena of the starting dead zone, chattering, stick-slip, a flat-topped, etc., which can considerably affect the system stability [46].

$$\begin{array}{ccc} T_{friction} & = & T_{friction1}+T_{friction2}+T_{friction3}+T_{friction4} \end{array}$$ (7)

**Fig 3:**
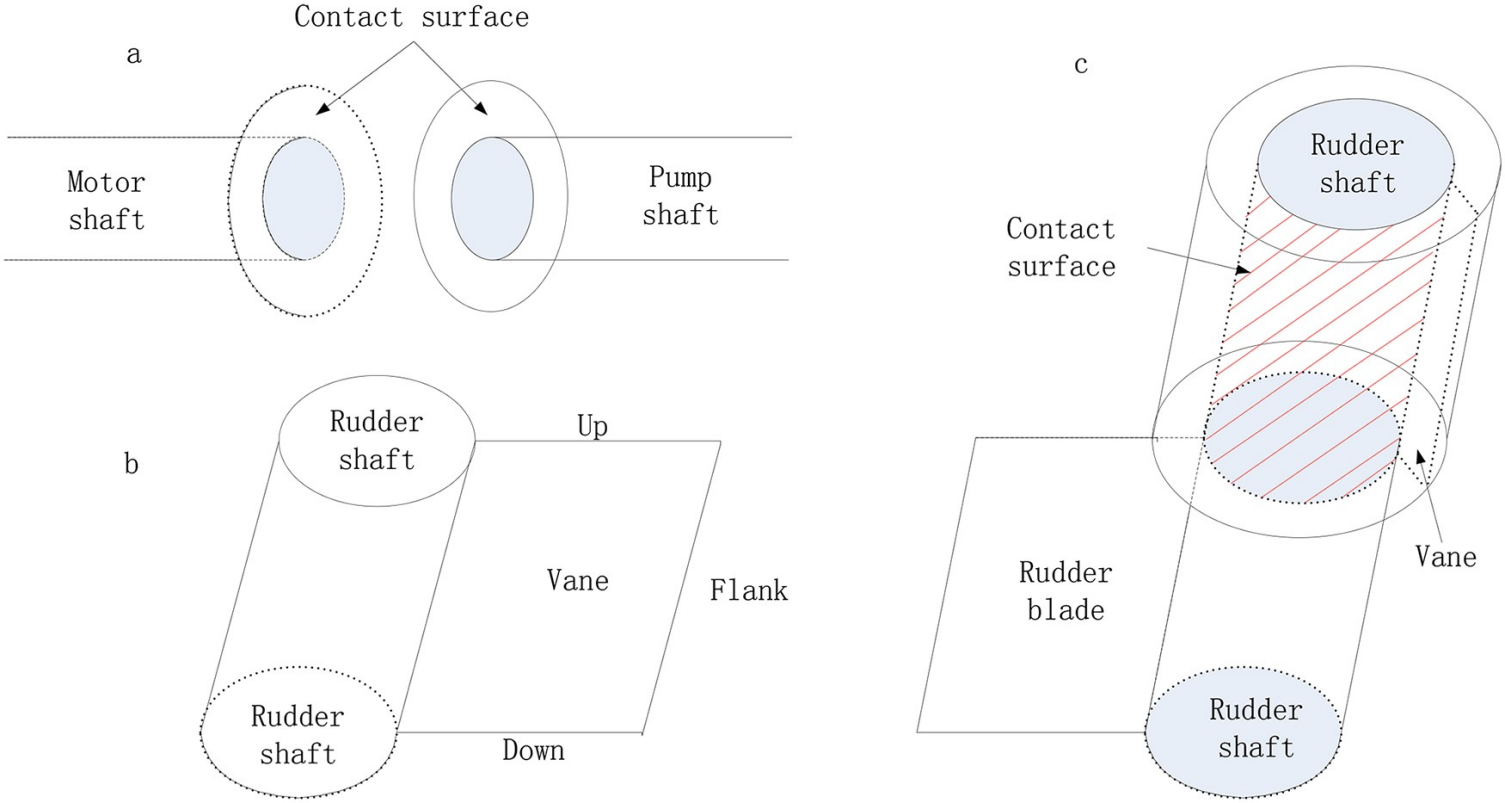
The surface-arisen friction.

The friction of the no-load state consists of the contact surface of coupling, which causes the phase difference between the motor shaft and the pump (Fig 3a), leading to the starting dead zone and flat-topping. The torque of the no-load steering gear caused by friction is described by Eq (8).

$$\begin{array}{c} \left\{ \begin{array}{c} T_{friction1}=\sigma_{0m}z_{m}+\sigma_{1m}\overset{•}{z_{m}}+B_{\theta_{m}}\overset{•}{\theta_{m}}\\ \overset{•}{z_{m}}=\overset{•}{\theta_{m}}-\frac{\left|\overset{•}{\theta_{m}}\right|}{g(\overset{•}{\theta_{m}})}z_{m}\\ \sigma_{0m}g\left(\overset{•}{\theta_{m}}\right)=T_{cm}+(T_{sm}-T_{cm})e^{-{\left[\frac{\overset{•}{\theta_{m}}}{\overset{•}{{\left(\theta_{m}\right)}_{s}}}\right]}^{2}} \end{array}\right. \end{array}$$ (8)

The friction-included actuator is in the surface between the vane and the oil chamber. The surface is divided into three parts: top, bottom, and flank. Fig 3b shows the sketch map of the vane stretched into the oil chamber. The friction of these contact surfaces often leads to the starting dead zone and the flat-topped phenomenon. The torque of the included actuator steering gear caused by friction is described by Eq (9).

$$\begin{array}{ccc} T_{friction2} & = & T_{frictionUP}+T_{frictionDOWN}+T_{frictionFLANK} \end{array}$$ (9)

The instant linear speed of each point on the up and down surfaces from the rudder shaft to the flank of the chamber is not the same and is proportional to the vane radius because of the vane rotation. The friction of the three surfaces is described by Eq (10).

$$\begin{array}{c} \left\{ \begin{array}{c} F_{fUP}=F_{fDOWN}=\int_{0}^{R}[\sigma_{0}z+\sigma_{1}\overset{•}{z}+B_{\theta 2}\left(\overset{•}{r\theta }\right)]dr\\ F_{fFLANK}=\sigma_{0}z+\sigma_{1}\overset{•}{z}+B_{\theta 2}\left(\overset{•}{R\theta }\right) \end{array}\right. \end{array}$$ (10)

The bristle average deformation of the surface, rotary speed of the steering gear, and friction torque equivalent to that of the rudder shaft from the servo system are denoted by z, $\overset{•}{\theta }$, and *T*_*friction*_, respectively. *T*_*friction*_ is described by the following model in Eq (11):

$$\begin{array}{c} \left\{ \begin{array}{c} T_{friction2}=2\int_{0}^{R}r[\sigma_{01}z_{1}+\sigma_{11}\overset{•}{z_{1}}+B_{\theta 2}\left(\overset{•}{r\theta }\right)]dr\\ +R[\sigma_{02}z_{2}+\sigma_{12}\overset{•}{z_{2}}+B_{\theta 2}\left(\overset{•}{R\theta }\right)]\\ \overset{•}{z_{1}}=\overset{•}{r\theta }-\frac{\left|\left(\overset{•}{r\theta }\right)\right|}{g(\overset{•}{r\theta })}z_{1},\overset{•}{z_{2}}=\overset{•}{R\theta }-\frac{\left|\left(\overset{•}{R\theta }\right)\right|}{g(\overset{•}{R\theta })}z_{2}\\ \sigma_{01}g\left(\overset{•}{r\theta }\right)=F_{c1}+(F_{s1}-F_{c1})e^{-{\left[\frac{\overset{•}{r\theta }}{\overset{•}{{\left(r\theta \right)}_{s}}}\right]}^{2}}\\ \sigma_{02}g\left(\overset{•}{R\theta }\right)=F_{c2}+(F_{s2}-F_{c2})e^{-{\left[\frac{\overset{•}{R\theta }}{\overset{•}{{\left(R\theta \right)}_{s}}}\right]}^{2}} \end{array}\right. \end{array}$$ (11)

The friction-included load exists in the surface between the rudder shaft and the outer cylinder wall (Fig 3c), leading to the starting dead zone and the flat-topped phenomenon. Eq (12) describes the torque of the included load steering gear caused by friction. The friction fringing onto load disturbance primarily depends on the external load, which will not be discussed in this paper.

$$\begin{array}{c} \left\{ \begin{array}{c} T_{friction3}=R_{r}\left[\sigma_{03}z_{3}+\sigma_{13}\overset{•}{z_{3}}+B_{\theta 3}\overset{•}{\left(R_{r}\theta \right)}\right]\\ \overset{•}{z_{3}}=\overset{•}{\left(R_{r}\theta \right)}-\frac{\left|\overset{•}{\left(R_{r}\theta \right)}\right|}{g\left(\overset{•}{\left(R_{r}\theta \right)}\right)}z_{3}\\ \sigma_{03}g\overset{•}{\left(R_{r}\theta \right)}=F_{c3}+(F_{s3}-F_{c3})e^{-{\left[\frac{\overset{•}{\left(R_{r}\theta \right)}}{{\overset{•}{\left(R_{r}\theta \right)}}_{s}}\right]}^{2}} \end{array}\right. \end{array}$$ (12)

This model assumes that *g*(*rθ*), *g*(*Rθ*) and *g*(*R*_*r*_ *θ*_*s*_) are constant for strictly positive real values only if they are bounded. Moreover, *σ*_0*n*_ (n = 1,2,3) and *σ*_1*n*_ (n = 1,2,3) are dynamic coefficients, while *F*_*cn*_(n = 1,2,3), *F*_*sn*_ (n = 1,2,3), and *B*_*θn*_(n = 1,2,3) are static coefficients.

The system is in a stable state when $\overset{•}{z_{n}}$ (n = 1,2,3) is equal to zero. The friction torque of system *T*_*ss*_, the rotary speed of steering gear $\overset{•}{\theta }$, and the static coefficient for the LuGre model have the following relationship:

$$\begin{array}{ccc} T_{ss} & = & T_{cm}+(T_{sm}-T_{cm})e^{-{\left[\frac{\overset{•}{\theta_{m}}}{\overset{•}{{\left(\theta_{m}\right)}_{s}}}\right]}^{2}}\text{sgn}\left(\overset{•}{\theta_{m}}\right)+B_{\theta m}\left(\overset{•}{\theta_{m}}\right)\\ & + & 2r[F_{c1}+(F_{s1}-F_{c1})e^{-{\left[\frac{\overset{•}{r\theta }}{\overset{•}{{\left(r\theta \right)}_{s}}}\right]}^{2}}\text{sgn}\left(\overset{•}{r\theta }\right)+B_{\theta 1}\left(\overset{•}{r\theta }\right)]\\ & + & R[F_{c2}+(F_{s2}-F_{c2})e^{-{\left[\frac{\overset{•}{R\theta }}{\overset{•}{{\left(R\theta \right)}_{s}}}\right]}^{2}}\text{sgn}\left(\overset{•}{R\theta }\right)+B_{\theta 2}\left(\overset{•}{R\theta }\right)]\\ & + & R_{r}\left[F_{c3}+(F_{s3}-F_{c3})e^{-{\left[\frac{\overset{•}{\left(R_{r}\theta \right)}}{\overset{•}{{\left(R_{r}\theta \right)}_{s}}}\right]}^{2}}\text{sgn}\left(\overset{•}{\left(R_{r}\theta \right)}\right)+B_{\theta 3}\left(\overset{•}{\left(R_{r}\theta \right)}\right)\right] \end{array}$$ (13)

Eq (13) shows that when the system is in a stable state, the LuGre model accurately describes the Stribeck phenomenon. The LuGre model depicts all the friction process’ static and dynamic characteristics with a first-order differential equation adapted for the design of friction compensation. The LuGre model is known because of its bristle model describing the random behavior of friction. The LuGre model is built using the average deformation based on the bristle model. Furthermore, the LuGre model is a continuous model that can provide a smooth transition among different friction states and is easy to use.

## Compensation methods

This section discusses further research on the identification and compensation method of the friction torque in the working process of the DDVC-FRVSG system.

As mentioned in the references, based on friction’s characteristics, the DDVC-FRVSG system always adopts two compensation methods: one that is independent of a model and one based on a model.

### Compensation-independent model

The high-gain PID method is one of the compensation methods independent of a model that aims to improve the system’s anti-jamming capability. The derivative term can improve the dynamic performance of the system under PID control. Derivative control cannot be used alone in engineering practice. First-order damping elements are added to the PID controller, which is a low-pass filter, to improve the system performance [46].

**Fig 4:**
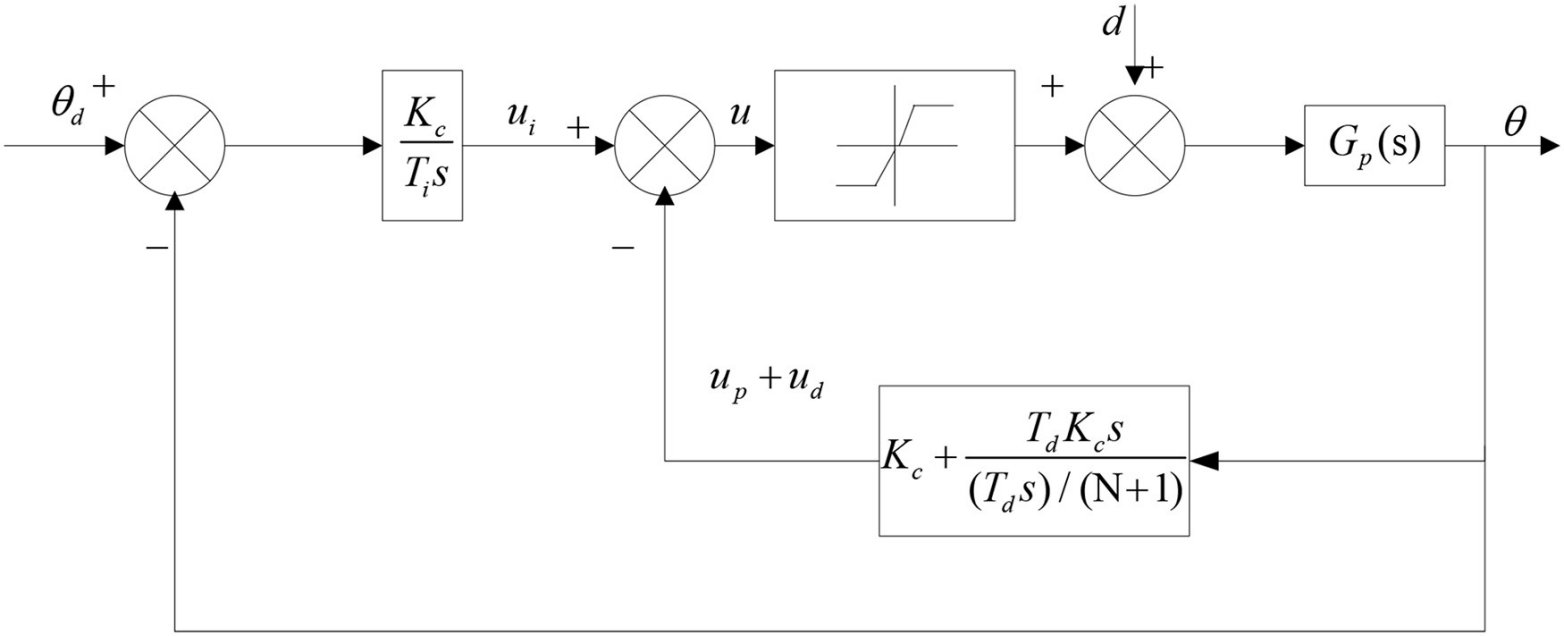
System chart of proportional derivative negative feedback.

Fig 4 depicts the system chart of the proportional derivative negative feedback. Using the proportional derivative negative feedback, for which the proportional element affects the rudder output only, the rudder output feedback after amplification by the proportional element is used to control the steering gear with the integral element. Therefore, a higher gain can be achieved, and overshoot and oscillation issues can be avoided using the structure of the proportional derivative negative feedback.

A higher closed-loop control gain must use a value of p that is adequately large to restrain the effect of friction and parameter changing. The real part of the closed-loop zero near the imaginary axis leads to the poor response of the closed-loop system by counteracting the effect of the pole.

A closed-loop zero is given as Eq (14).

$$\begin{array}{c} s=-\frac{N}{T_{d}}=-5p+2\omega_{h}\xi_{h} \end{array}$$ (14)

The only closed-loop zero is five times the closed-loop pole. The influence of the closed-loop system determined by the pole is weak, and the effects are negligible. Therefore, relative to the normal PID control structure, the performance of the proportional derivative negative feedback PID control system is only determined by the closed-loop pole using a higher closed-loop gain. The closed-loop poles are assigned as multiple poles on the negative real axis to ensure that the response of the closed-loop system is significantly enhanced, thereby restraining friction and improving the system performance.

### Compensation based on the model

The following assumptions are first given to design the self-adaptation robust control of the DDVC-FRVSG system nonlinear friction [47]:

Assumption 1: the scope of parameter uncertainty is known as Eq (15).

$$\begin{array}{c} X\in \Omega ≜\{X:0<X_{\min}\leq X\leq X_{\max}\} \end{array}$$ (15)

where, *X*_min_ = [*X*_1 min_, *X*_2 min_, …, *X*_*n* min_]^*T*^, *X*_max_ = [*X*_1 max_, *X*_2 max_, …, *X*_*n* max_]^*T*^.

Assumption 2: the scope of nonlinear uncertainty Δ has been determined by the product of the known positive function *δ*(*x*, *t*) and the unknown run-time function *d*(*t*) as Eq (16)

$$\begin{array}{c} {∥\Delta (x,z,u,t)∥}_{\infty }\leq \delta \left(x,t\right)\cdot {∥d(t)∥}_{\infty } \end{array}$$ (16)

where, ∥⋅∥_*∞*_ indicates the *L*_∞_ standard. Given that *θ*_*d*_(*t*) is the given track, the control target is to input *u*, such that the system can enable the system output *θ*(*t*) to track *θ*_*d*_(*t*) as far as possible under different modeling uncertainties.

Given that *z*(0) indicates the average deformation of bristle at the initial moment, and *z*(*t*) indicates the average deformation of bristle at any moment t, as it can be seen from the LuGre model described in Eqs (7)–(13), the bristle deformation is featured with the following based on literature [26]: if |*z*(0)| ≤ *T*_*sf*_, then |*z*(*t*)| ≤ *T*_*sf*_.

The sliding mode surface is redefined as Eq (17) based on the aforesaid assumptions.

$$\begin{array}{ccc} e_{2} & = & \overset{\cdot }{e_{1}}\;+ke_{1}=\overset{\cdot }{\theta }\;-\theta_{2eq}\\ \theta_{2eq} & = & \overset{\cdot }{\theta_{d}}\;-ke_{1} \end{array}$$ (17)

In such case, the problem in tracking *θ*_*d*_ is minimized *e*_2_. The self-adaptation robust controller proposed is shown as Eq (18).

$$\begin{array}{c} u=u_{a}+u_{s1}+u_{s2} \end{array}$$ (18)

where, *u*_*a*_ indicates the compensation term of the friction model; *u*_*s*1_ indicates the robust feedback term; and *u*_*s*2_ indicates the robust control term.

The compensation term of the friction model *u*_*a*_ can be designed as Eq (19).

$$\begin{array}{c} u_{a}=\frac{1}{a}[b\overset{\cdot }{\theta }\;+\overset{\wedge }{\sigma_{0}}\;\overset{\wedge }{z_{0}}\;-\overset{\wedge }{\sigma_{1}}\;\frac{\left|\overset{\cdot }{\theta }\;\right|}{g(\overset{\cdot }{\theta }\;)}\overset{\wedge }{z_{1}}\;+\overset{\wedge }{\beta }\;\overset{\cdot }{\theta }\;+c\overset{\cdot }{T}\;+J{\overset{\cdot }{\theta }\;}_{2eq}] \end{array}$$ (19)

where, $\overset{\wedge }{\sigma_{0}}\;$, $\overset{\wedge }{\sigma_{1}}\;$, and $\overset{\wedge }{\beta }\;$ indicates the estimated value of the unknown parameters.

The state variable *z* of the LuGre friction model also adopts two nonlinear observers for independent description, as shown in Eq (20).

$$\begin{array}{ccc} \frac{d\overset{\wedge }{z_{0}}\;}{dt} & = & \overset{\cdot }{\theta }\;-\frac{\left|\overset{\cdot }{\theta }\;\right|}{g(\overset{\cdot }{\theta }\;)}\overset{\wedge }{z_{0}}\;-\gamma_{0}e_{2}\\ \frac{d\overset{\wedge }{z_{1}}\;}{dt} & = & \overset{\cdot }{\theta }\;-\frac{\left|\overset{\cdot }{\theta }\;\right|}{g(\overset{\cdot }{\theta }\;)}\overset{\wedge }{z_{1}}\;-\gamma_{1}\frac{\left|\overset{\cdot }{\theta }\;\right|}{g(\overset{\cdot }{\theta }\;)}\overset{\wedge }{e_{2}} \end{array}$$ (20)

where, *γ*_0_ and *γ*_1_ are two positive design parameters.

In this case, *X* = [*σ*_0_, *σ*_1_, *β*] = [*x*_1_, *x*_2_, *x*_3_], the self-adaptation law of $\varphi^{T}=[-\overset{\wedge }{z_{0}}\;,(|\overset{\cdot }{\theta }\;|/g(\overset{\cdot }{\theta }\;)\overset{\wedge }{z_{1}}\;,),-\overset{\cdot }{\theta }\;]$ parameter adopts the following forms as Eq (21):

$$\begin{array}{c} \overset{\cdot }{\overset{\wedge }{X}\;}=\Gamma \varphi e_{2} \end{array}$$ (21)

where, $\overset{\wedge }{X}\;(0)$ (the initial value of the parameter) is the nominal value *X*_*nom*_ of the parameter, and Γ refers to the self-adaptation law array.

A discontinuity projection map method is adopted to convert the non-linear system with friction into the semi strictly feedback mode and solve the problem in robust tracking control. The $\overset{\wedge }{X}\;$ parameter is converted into the following iterative formulas, as Eq (22):

$$\begin{array}{c} \overset{\cdot }{\overset{\wedge }{X}\;}\;=\mathrm{Pr}oj_{X}\left(\Gamma \varphi e_{2}\right) \end{array}$$ (22)

where, in $\mathrm{Pr}oj_{X}\left(\Gamma \varphi e_{2}\right)={\left[\mathrm{Pr}oj_{X_{1}}{\left(\Gamma \varphi e_{2}\right)}_{1},...,\mathrm{Pr}oj_{X_{3}}{\left(\Gamma \varphi e_{2}\right)}_{3}\right]}^{T}$, (Γ*φe*_2_)_*i*_ indicates the *i*^*th*^ item of Γ*φe*_2_. The unmeasured friction state z can be converted into the following robust observer to map the estimates:

$$\begin{array}{ccc} \frac{d\overset{\wedge }{z_{0}}\;}{dt} & = & \mathrm{Pr}oj_{s0}\{\overset{\cdot }{\theta }\;-\frac{\left|\overset{\cdot }{\theta }\;\right|}{g(\overset{\cdot }{\theta }\;)}\overset{\wedge }{z_{0}}\;-\gamma_{0}e_{2}\} \end{array}$$ (23)

$$\begin{array}{ccc} \frac{d\overset{\wedge }{z_{1}}\;}{dt} & = & \mathrm{Pr}oj_{s1}\{\overset{\cdot }{\theta }\;-\frac{\left|\overset{\cdot }{\theta }\;\right|}{g(\overset{\cdot }{\theta }\;)}\overset{\wedge }{z_{1}}\;-\gamma_{1}\frac{\left|\overset{\cdot }{\theta }\;\right|}{g(\overset{\cdot }{\theta }\;)}e_{2}\} \end{array}$$ (24)

The projection map is presented as follows in Eqs (22) ~ (24):

$$\begin{array}{c} \mathrm{Pr}oj_{\zeta }\;(f)=\{\begin{array}{cccc} 0 & , & if\overset{\wedge }{\zeta }\;=\zeta_{\max}, & f>0\\ 0 & , & if\overset{\wedge }{\zeta }\;=\zeta_{\min}, & f<0\\ f & , & other \end{array} \end{array}$$ (25)

where, *f* indicates the self-adaptation law function of the given parameter, and *ζ* refers to *σ*_0_, *σ*_1_, *β*, *z*_0_, or *z*_1_. For example, Eq (23) can be written in the form of Eq (26).

$$\begin{array}{c} \frac{d\overset{\wedge }{z_{0}}\;}{dt}=\{\begin{array}{cccc} 0 & , & if\overset{\wedge }{z_{0}}\;=z_{0\max}, & \overset{\cdot }{\theta }\;-\frac{\left|\overset{\cdot }{\theta }\;\right|}{g(\overset{\cdot }{\theta }\;)}\overset{\wedge }{z_{0}}\;-\gamma_{0}e_{2}>0\\ 0 & , & if\overset{\wedge }{z_{0}}\;=z_{0\min}, & \overset{\cdot }{\theta }\;-\frac{\left|\overset{\cdot }{\theta }\;\right|}{g(\overset{\cdot }{\theta }\;)}\overset{\wedge }{z_{0}}\;-\gamma_{0}e_{2}<0\\ \overset{\cdot }{\theta }\;-\frac{\left|\overset{\cdot }{\theta }\;\right|}{g(\overset{\cdot }{\theta }\;)}\overset{\wedge }{z_{0}}\;-\gamma_{0}e_{2}>0 & , & other \end{array} \end{array}$$ (26)

The aforesaid projection map has characteristics based on literature [45] and as shown in Eq (27).

$$\begin{array}{cc} & \zeta_{\min}\leq \overset{\wedge }{\zeta }\;\leq \zeta_{\max}\\ & {\tilde{X}\;}^{T}(\Gamma^{-1}\mathrm{Pr}oj_{X}\left(\Gamma \varphi e_{2}\right)-\varphi e_{2})\leq 0\\ & \tilde{z_{0}}\;\{\mathrm{Pr}oj_{s0}(\overset{\cdot }{\theta }\;-\frac{\left|\overset{\cdot }{\theta }\;\right|}{g(\overset{\cdot }{\theta }\;)}\overset{\wedge }{z_{0}}\;-\gamma_{0}e_{2})-(\overset{\cdot }{\theta }\;-\frac{\left|\overset{\cdot }{\theta }\;\right|}{g(\overset{\cdot }{\theta }\;)}\overset{\wedge }{z_{0}}\;-\gamma_{0}e_{2})\}\leq 0\\ & \tilde{z_{1}}\;\{\mathrm{Pr}oj_{s1}(\overset{\cdot }{\theta }\;-\frac{\left|\overset{\cdot }{\theta }\;\right|}{g(\overset{\cdot }{\theta }\;)}\overset{\wedge }{z_{1}}\;+\gamma_{1}\frac{\left|\overset{\cdot }{\theta }\;\right|}{g(\overset{\cdot }{\theta }\;)}e_{2})-(\overset{\cdot }{\theta }\;-\frac{\left|\overset{\cdot }{\theta }\;\right|}{g(\overset{\cdot }{\theta }\;)}\overset{\wedge }{z_{2}}\;+\gamma_{1}\frac{\left|\overset{\cdot }{\theta }\;\right|}{g(\overset{\cdot }{\theta }\;)}e_{2})\}\leq 0 \end{array}$$ (27)

The robust feedback term *u*_*s*1_ is constructed as Eq (28).

$$\begin{array}{c} u_{s1}=-\frac{k_{s}}{a}e_{2} \end{array}$$ (28)

where, *k*_*s*_ refers to the positive feedback gain.

The robust control term *u*_*s*2_ is constructed as Eq (29).

$$\begin{array}{c} J\frac{de_{2}}{dt}=-k_{s}e_{2}-\varphi^{T}\tilde{X}\;-\sigma_{0}\tilde{z_{0}}\;+\sigma_{1}\frac{\left|\overset{\cdot }{\theta }\;\right|}{g(\overset{\cdot }{\theta }\;)}\tilde{z_{1}}\;+au_{s2}+\Delta \end{array}$$ (29)

where, $\tilde{X}\;=\overset{\wedge }{X}\;-X$ refers to the parameter estimation error. The self-adaptation robust friction compensation control strategy weakens the impact of the model uncertainty by *u*_*s*1_ and *u*_*s*2_ to realize the ideal control performance. Based on assumptions 1 and 2, *u*_*s*2_ must meet the conditions as Eq (30).

$$\begin{array}{c} e_{2}[-\varphi^{T}\tilde{X}\;-\sigma_{0}\tilde{z_{0}}\;+\sigma_{1}\frac{\left|\overset{\cdot }{\theta }\;\right|}{g(\overset{\cdot }{\theta }\;)}\tilde{z_{1}}\;+au_{s2}]\leq \varepsilon_{0}+\varepsilon_{1}{\left|∥d∥\right|}_{\infty }^{2} \end{array}$$ (30)

where, *ε*_0_ and *ε*_1_ are two design parameters. The conditions in Eq (31) must be met to ensure that the self-adaptation and friction state estimation have no effect on the robust control term *u*_*s*2_ for the parameter.

$$\begin{array}{c} e_{2}au_{s2}\leq 0 \end{array}$$ (31)

A *u*_*s*2_ meeting the requirements of Eqs (30)) and (31) can be given by the mode of Eq (32). Given that *h*_0_, *h*_1_, and *h*_2_ are the smooth condition and boundedness function meeting the following conditions [48, 49]:

$$\begin{array}{ccc} h_{0} & \geq & {∥\varphi ∥}_{2}\cdot {∥X_{\max}-X_{\min}∥}_{2}\\ h_{1} & \geq & \sigma_{0\max}(Z_{\max}-Z_{\min})\\ h_{2} & \geq & \sigma_{1\max}\frac{\left|\overset{\cdot }{\theta }\;\right|}{g(\overset{\cdot }{\theta }\;)}(Z_{\max}-Z_{\min}) \end{array}$$ (32)

where, ∥⋅∥_2_ indicates a Euclid canonical form. *φ* and |*x*_2_|/*g*(*x*_2_) require the real measured signal, and *h*_1_ and *h*_2_ need real-time computing. In such a case, *u*_*s*2_ can be represented by the following functions:

where *h*_0_, *h*_1_ and *h*_2_ can be taken as Eq (33).

$$\begin{array}{ccc} h_{0} & = & {∥\varphi ∥}_{2}\cdot {∥X_{\max}-X_{\min}∥}_{2}\\ h_{1} & = & \sigma_{0\max}(Z_{\max}-Z_{\min})\\ h_{2} & = & \sigma_{1\max}\frac{\left|\overset{\cdot }{\theta }\;\right|}{g(\overset{\cdot }{\theta }\;)}(Z_{\max}-Z_{\min}) \end{array}$$ (33)

The design of the friction self-adaptation robust compensation controller has so far been completed.

As proven by the asymptotic stability, the Lyapunov function of Eq (34) shall be considered.

$$\begin{array}{c} V_{s}=\frac{1}{2}J{e_{2}}^{2} \end{array}$$ (34)

Eq (35) can be obtained from Eqs (29) and (30).

$$\begin{array}{ccc} \overset{\cdot }{V_{s}}\; & = & -k_{s}e_{2}^{2}+e_{2}[-\varphi^{T}\tilde{X}\;+\sigma_{0}\tilde{z_{0}}\;-\sigma_{1}\frac{\left|\overset{\cdot }{\theta }\;\right|}{g(\overset{\cdot }{\theta }\;)}\tilde{z_{1}}\;+\Delta +au_{s2}]\\ & \leq & -\lambda_{V}V_{s}+\varepsilon_{0}+\varepsilon_{1}{∥d∥}_{\infty }^{2} \end{array}$$ (35)

Inferring as Eq (36),

$$\begin{array}{c} V_{s}\leq \exp(-\lambda_{V}t)V_{s}(0)+\frac{\varepsilon_{0}+\varepsilon_{1}{∥d∥}_{\infty }^{2}}{\lambda_{V}}[1-\exp(-\lambda_{V}t)] \end{array}$$ (36)

where, λ_*V*_ = 2*k*_*s*_/*J*. As a result, *e*_2_(*t*) can be known as bounded. *e*_1_(*t*) and *e*_2_(*t*) can meet the requirements of the exponentially stable transfer function *G*(*s*) = (1/(*s* + *k*)); therefore, *e*_1_(*t*) is bounded. *x*_*d*_(*t*) is the bounded signal of a bounded second derivative; hence, Eq (17) infers that *x*_*d*_(*t*) is bounded. *e*_1_ = *x*_1_ − *x*_*d*_, *e*_2_ = *x*_2_ − *x*_2*eq*_; thus, the state vector *x* is also bounded. Eq (27) inferes that $\overset{\wedge }{\theta }\;$, $\overset{\wedge }{z_{0}}\;$ and $\overset{\wedge }{z_{1}}\;$ are bounded. Therefore, the control input u is bounded, indicating that the controller proposed herein can obtain the given transient performance and tracking accuracy.

The Lyapunov function of Eq (37) shall be considered.

$$\begin{array}{c} V_{a}=\frac{1}{2}J{e_{2}}^{2}+\frac{1}{2\gamma_{0}}+\sigma_{0}{\tilde{z_{0}}\;}^{2}+\frac{1}{2\gamma_{1}}+\sigma_{1}{\tilde{z_{1}}\;}^{2}+\frac{1}{2}{\tilde{X}\;}^{T}\Gamma^{-1}\tilde{X}\; \end{array}$$ (37)

Eq (29) shows its derivative in Eq (38).

$$\begin{array}{c} \overset{\cdot }{V_{a}}\;=-k_{s}e_{2}^{2}+e_{2}[-\varphi^{T}\tilde{X}\;+\sigma_{0}\tilde{z_{0}}\;-\sigma_{1}\frac{\left|\overset{\cdot }{\theta }\;\right|}{g(\overset{\cdot }{\theta }\;)}\tilde{z_{1}}\;+au_{s2}]+\frac{\sigma_{0}}{\gamma_{0}}\tilde{z_{0}}\;(\overset{\cdot }{\overset{\wedge }{z_{0}}\;}\;-\overset{\cdot }{z}\;) \end{array}$$ (38)

Eq (39) can be obtained from Eq (31).

$$\begin{array}{ccc} \overset{\cdot }{V_{a}}\; & \leq & -k_{s}e_{2}^{2}+\sigma_{0}\tilde{z_{0}}\;(\frac{\overset{\cdot }{\overset{\wedge }{z_{0}}\;}\;}{\gamma_{0}}+e_{2})-\frac{\sigma_{0}}{\gamma_{0}}\tilde{z_{0}}\;\overset{\cdot }{z}\;+\sigma_{1}\tilde{z_{1}}\;(\frac{\overset{\cdot }{\overset{\wedge }{z_{1}}\;}\;}{\gamma_{1}}-\frac{\left|\overset{\cdot }{\theta }\;\right|}{g(\overset{\cdot }{\theta }\;)}e_{2})\\ & - & \frac{\sigma_{1}}{\gamma_{1}}\tilde{z_{1}}\;\overset{\cdot }{z}\;+\tilde{X^{T}}\;(\Gamma^{-1}\overset{\cdot }{\overset{\wedge }{X}\;}\;-\varphi e_{2}) \end{array}$$ (39)

Substitute Eqs (22), (23) and (24) into Eq (39) and obtain Eq (40).

$$\begin{array}{ccc} \overset{\cdot }{V_{a}}\; & \leq & -k_{s}e_{2}^{2}+\frac{\sigma_{0}}{\gamma_{0}}\tilde{z_{0}}\;(\overset{\cdot }{\overset{\wedge }{z_{0}}\;}\;-\overset{\cdot }{\theta }\;+\frac{\left|\overset{\cdot }{\theta }\;\right|}{g(\overset{\cdot }{\theta }\;)}\overset{\wedge }{z_{0}}\;+\gamma_{0}e_{2})-\frac{\sigma_{0}}{\gamma_{0}}\tilde{z_{0}}\;(\overset{\cdot }{z}\;-\overset{\cdot }{\theta }\;+\frac{\left|\overset{\cdot }{\theta }\;\right|}{g(\overset{\cdot }{\theta }\;)}\overset{\wedge }{z_{0}}\;)\\ & + & \frac{\sigma_{1}}{\gamma_{1}}\tilde{z_{1}}\;(\overset{\cdot }{\overset{\wedge }{z_{1}}\;}\;-\overset{\cdot }{\theta }\;+\frac{\left|\overset{\cdot }{\theta }\;\right|}{g(\overset{\cdot }{\theta }\;)}\overset{\wedge }{z_{1}}\;-\gamma_{1}\frac{\left|\overset{\cdot }{\theta }\;\right|}{g(\overset{\cdot }{\theta }\;)}e_{2})-\frac{\sigma_{1}}{\gamma_{1}}\tilde{z_{1}}\;(\overset{\cdot }{z}\;-\overset{\cdot }{\theta }\;+\frac{\left|\overset{\cdot }{\theta }\;\right|}{g(\overset{\cdot }{\theta }\;)}\overset{\wedge }{z_{1}}\;)\\ & + & \tilde{X^{T}}\;(\Gamma^{-1}\overset{\cdot }{\overset{\wedge }{X}\;}\;-\varphi e_{2}) \end{array}$$ (40)

Eq (40) can be simplified into Eq (41) as follows based on Eq (27):

$$\begin{array}{c} \overset{\cdot }{V_{a}}\;\leq -k_{s}e_{2}^{2}-\frac{\sigma_{0}}{\gamma_{0}}\tilde{z_{0}}\;(\overset{\cdot }{z}\;-\overset{\cdot }{\theta }\;+\frac{\left|\overset{\cdot }{\theta }\;\right|}{g(\overset{\cdot }{\theta }\;)}\overset{\wedge }{z_{0}}\;)-\frac{\sigma_{1}}{\gamma_{1}}\tilde{z_{1}}\;(\overset{\cdot }{z}\;-\overset{\cdot }{\theta }\;+\frac{\left|\overset{\cdot }{\theta }\;\right|}{g(\overset{\cdot }{\theta }\;)}\overset{\wedge }{z_{1}}\;) \end{array}$$ (41)

Use Eq (7) to substitute *z*, such that the above formula can be converted into Eq (42).

$$\begin{array}{c} \overset{\cdot }{V_{a}}\;\leq -k_{s}e_{2}^{2}-\frac{\sigma_{0}}{\gamma_{0}}\tilde{\frac{\left|\overset{\cdot }{\theta }\;\right|}{g(\overset{\cdot }{\theta }\;)}{\tilde{z_{0}}\;}^{2}-\frac{\sigma_{1}}{\gamma_{1}}\frac{\left|\overset{\cdot }{\theta }\;\right|}{g(\overset{\cdot }{\theta }\;)}{\tilde{z_{1}}\;}^{2}\leq }\;-k_{s}e_{2}^{2} \end{array}$$ (42)

Therefore, *e*_2_ ∈ *L*_2_ ∩ *L*_∞_. $\overset{\cdot }{e_{2}}\;\in L_{\infty }$ can be obtained from Eq (29). Based on Barbalat theorem, when *t* → ∞, *e*_2_ → 0. *e*_1_(*t*) and *e*_2_(*t*) meet the relationship of the exponentially stable transfer function; hence, $\underset{t\to \infty }{\text{lim}}e_{1}\left(t\right)=0$ can be inferred, ensuring *θ* to asymptotically converge to *θ*_*d*_.

The abovementioned process makes use of the Lyapunov-based method and proves the asymptotic stability of the designed self-adaptation robust dynamic friction compensation control method.

## Results

### Test bench

The main hardware used in this work consists of an AC servomotor, a motor drive, a coupling, a gear pump, a pre-pressing oil tank, a support device, a rotary vane steering gear, a rudder blade, a feedback device, a semi-physical simulation platform of quanser, and a control computer.Table 2 lists the parameter for the test bench.

**Table 2:** Parameter of test bench.

Parameter Name	Quantity	Units
Servo motor rated power	15	*Kw*
Servo motor rated speed	1460	*rev*/*min*
Diameter of coupling	0.12	*m*
Displacement of gear pump	3.3 × 10^−5^	*m*^3^/*rev*
Volume of pre-pressing oil tank	0.01	*m*^3^
The inner diameter of the outer cylinder	0.18	*m*
The outer diameter of the inner cylinder	0.14	*m*
Height of rotary vane	0.205	*m*
Width of stator and rotary vane	0.0375	*m*
Area of rudder blade	4.2	*m*^2^
Equilibrium ratio of rudder blade	0.35	zero dimension
Aspect ratio of rudder blade	1.5	zero dimension
Amplification coefficient of angle sensor	16.38	zero dimension

A quanser is a mechatronic integration tool that offers a suite of third-party device blocks that help researchers seamlessly interface and control devices. These blocks not only allow a Simulink model to communicate with external devices, but also implement the mathematical framework for controlling them. In a test, the system framework comprised the quanser modules in the Simulink. Through the quanser semi-physical simulation platform, analog voltage was transmitted to the servomotor driver to control the motor’s speed. When the motor rotated, it drove the gear pump, and the pump, in turn, rotated the vane cylinder. The cylinder rotated the rudder blade. The parameters of the rudder angle were collected via the rudder angle feedback device. A sensor changed the parameters into simulated voltage signals. The signals were changed into digital signals and transmitted to a computer through the semi-physical simulation platform.

### Experimental results

**Fig 5:**
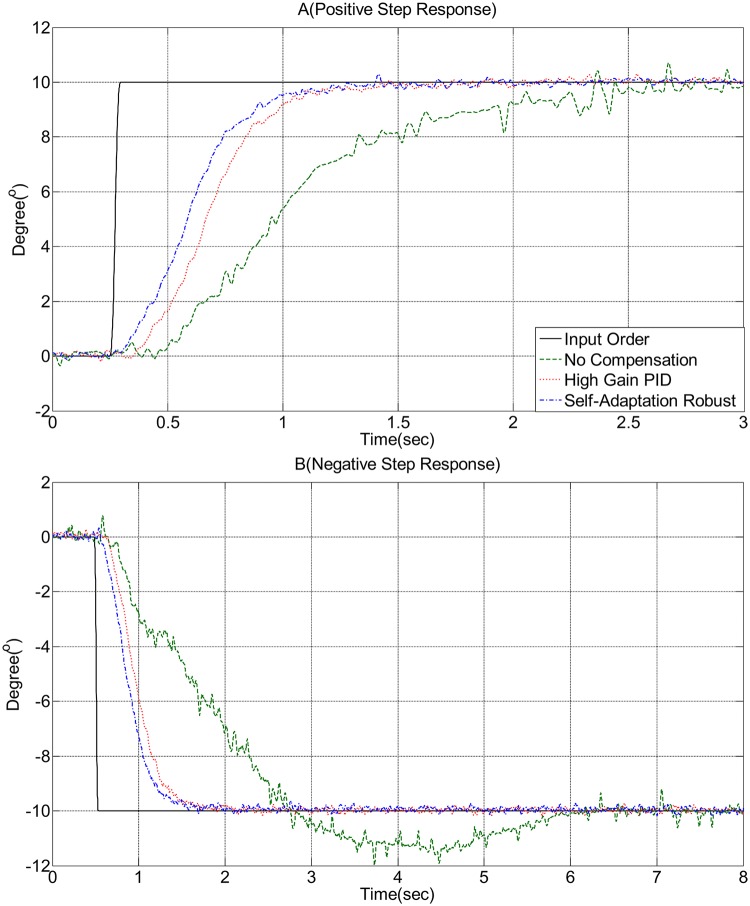
Experimental result: Step response.

**Fig 6:**
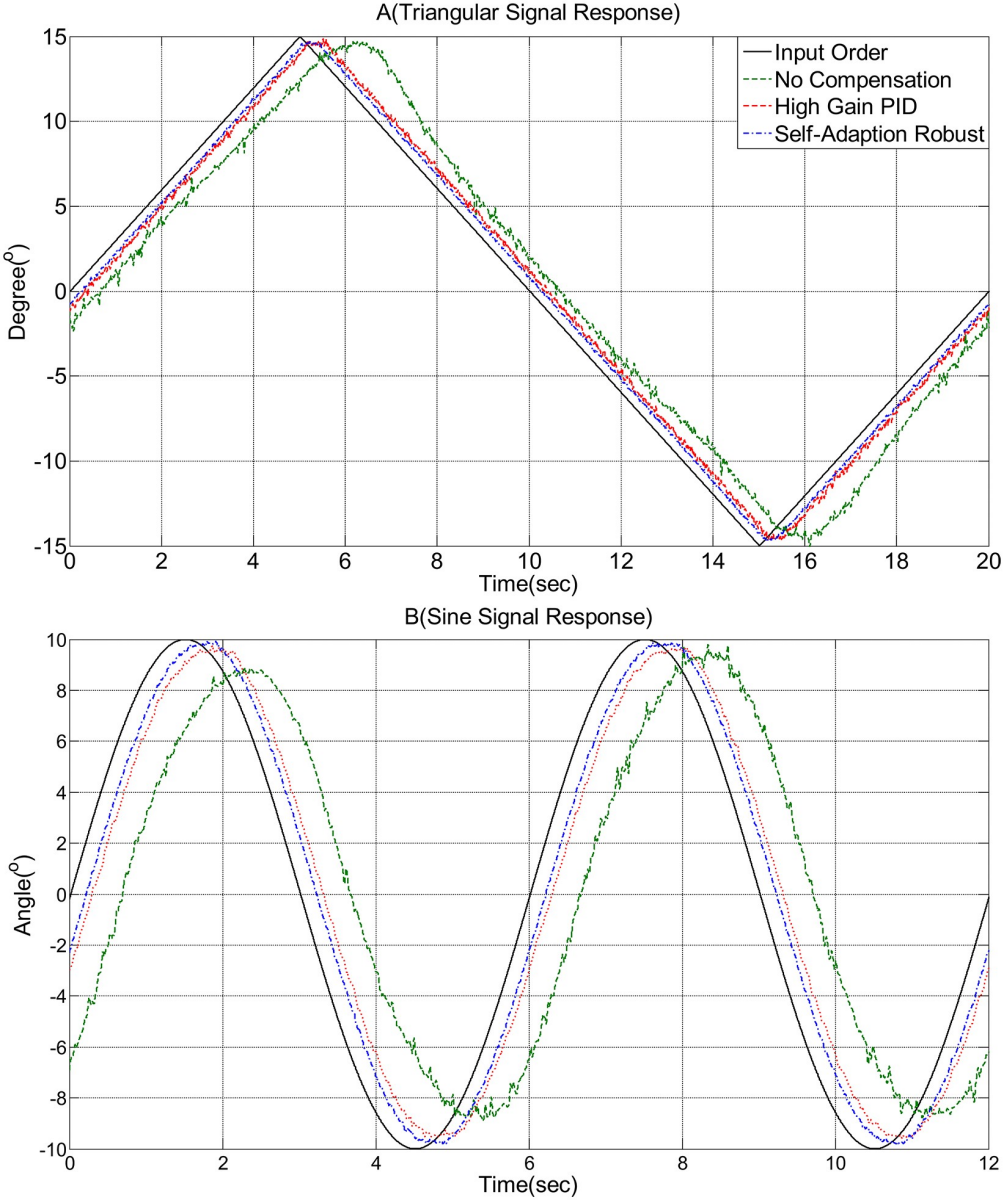
Experimental result: Triangular and sine signal response.

Figs 5 and 6 show the test results of the rotary vane steering gear under non-compensation control, high-gain PID control, and self-adaption robust control. The full line represents the angle input order. The long dashed line represents the non-compensation control, while the short dashed line represents the high-gain PID control. The dash-and-dot line represents the self-adaption robust control.

Fig 5A shows the steering gear driven via a positive step signal. In the start up, when the value of the output torque from the rotary vane cylinder to the rudder blade (output) is less than the value of the maximum static friction torque, the rudder blade begins to exhibit a starting dead zone and a chattering phenomenon (e.g., long dashed line from 0.23 s to 0.48 s that continued for 0.25 s), as shown in Fig 5A. The reason for the starting dead zone phenomenon is that in the starting procedure, the system needs to overcome the maximum static friction torque, which causes the chattering phenomenon when the output torque is close to the maximum static friction torque, as shown in the figure in the starting dead zone areas. The starting dead zone phenomenon present in the high-gain PID state was reduced to a certain extent. The dead zone from 0.23 s to 0.38 s, which continued to 0.15 s (short dotted line, Fig 5A), was considerably shortened compared to the long dashed line (i.e., reduced by 40%). The starting dead zone phenomenon present in the no-compensation state and the high-gain PID compensation state considerably decreased. The dead zone from 0.23 s to 0.302 s, which continued to 0.072 s (dash-and-dot line, Fig 5A) considerably shortened compared to the short dotted line and reduced by 52%. Moreover, a further substantial reduction compared to the long dashed line showed a reduction of 71.2%. The value of the error decreased when the rudder angle approached the input order. The rotary speed decreased, and the output torque of the rotary vane cylinder gradually decreased, showing a repeated shift between the dynamic and static frictions. Repeating the above mentioned process, the rudder blade began to exhibit a stick-slip phenomenon (long dashed line from 0.48 s to 0.99 s and 1.33 s to 2.63 s that continued to 1.81 s in Fig 5A). The stick-slip phenomenon present in the high-gain PID state from 0.38 s to 0.6 s and 0.81 s to 1.62 s, which continued to 1.03 s (short dotted line, Fig 5A), was considerably shortened compared to the long dashed line (i.e., reduced by 43.1%). The phenomenon present in the self-adaption robust state from 0.302 s to 0.5 s and 0.71 s to 1.22 s, which continued to 0.728 s (dash-and-dot line, Fig 5A) considerably shortened compared to the short dotted line (i.e., reduced by 29.3%). A further substantial reduction compared to the long dashed line showed a reduction of 59.8%.Table 3 lists the result for the experimental.

**Table 3:** Duration of positive step response.

	No-compensation	High-gain PID	Self-adaption robust
*Deadzone*	0.250s	0.150s	0.072s
*Chattering*	0.250s	0.150s	0.072s
*Stick*–*slip*	1.810s	1.030s	0.728s

Fig 5B shows the steering gear driving by a negative step signal. The starting dead zone phenomena present in the high-gain PID state showed a reduction of 40% compared to the no-compensation state. The phenomenon present in the self-adaption robust state reduced by 54.5% compared to the high-gain PID state and by 72.7% compared to the no-compensation state. The chattering phenomena present in the high-gain PID state showed a reduction of 43.3% compared to the no-compensation state. The amplitude of chattering reduced by 85.7%. The phenomenon present in the self-adaption robust state reduced by 76.3% compared to the high-gain PID state and by 86.5% compared to the no-compensation state. The stick-slip phenomenon present in the high-gain PID state reduced by 37.9% compared to the no-compensation state. The phenomenon present in the self-adaption robust state reduced by 34.3% compared to the high-gain PID state and by 59.2% compared to the no-compensation state.Table 4 lists the result for the experimental.

**Table 4:** Duration of negative step response.

	No-compensation	High-gain PID	Self-adaption robust
*Deadzone*	0.275s	0.165s	0.075s
*Chattering*	0.275s	0.156s	0.037s
*Stick*–*slip*	1.740s	1.080s	0.710s

Fig 6A and 6B show the steering gear driven by a triangular wave signal and a sine wave signal, respectively. The rotary direction changed because of the rudder blade operating in a low-velocity state. Therefore, the flat-topped phenomenon was exhibited during almost the entire process of changing direction (long dashed line, Fig 6A and 6B). The short dotted line in Fig 6A and 6B shows that the duration was considerably shortened, and the curve tended to be smooth compared to the no-compensation state. The duration of the flat-topped phenomenon present in the no-compensation state was shortened in the process of changing direction (short dotted line, Fig 6A and 6B). The dash-and-dot line in Fig 6A and 6B depict that the duration of the flat-topped phenomenon present in the no-compensation state and compensated by the high-gain PID state was almost eliminated in the process of changing direction (dash-and-dot line, Fig 6A and 6B). Table 5 lists the result for the experimental.

**Table 5:** Duration of triangular wave(A) and sine wave(B) response.

	No-compensation	High-gain PID	Self-adaption robust
*Flat*–*topped*(*A*)	1.780s	1.080s	0.770s
*Stick*–*slip*(*B*)	9.400s	5.905s	5.106s
*Flat*–*topped*(*B*)	2.610s	2.080s	1.276s

## Conclusion

This study combined concepts of electro-hydraulic engineering, identification techniques, and compensation methods to design a DDVC-FRVSG system controller. We proposed two compensation methods to suppress the nonlinear rudder friction and effectively and precisely control the system. We conducted a rudder angle response test for the DDVC-FRVSG under three states: non-compensation control, high-gain PID control, and self-adaption robust control. The study makes a significant contribution to the literature because both methods can compensate for the nonlinear friction, with the second strategy showing a better performance of up to 78.85% compared to the 41.65% increase shown by the first strategy. Therefore, the self-adaption robust compensation control method effectively suppresses the nonlinear friction torque and ensures the stable operation of the DDVC-FRVSG.

The self-adaption robust control compensation designed herein improved the DDVC-FRVSG by up to 78.85% compared to the no-compensation state. However, the nonlinear phenomena of the starting dead zone, stick-slip, and flat-topped were not eliminated; thus, the dynamic performance still has the potential to improve. [50–55] proposed a compensation method based on the Lyapunov theory to compensate for the nonlinear friction during the operation of a ball-screw electro-mechanical servo system. They used the LuGre model to identify the parameters of the friction model online and designed a compensation controller through an adaptive back-stepping control method that obtained ideal compensation effects.

Our future work will apply an adaptive back-stepping control method to the DDVC-FRVSG. Some similarities exist in operation; however, compared to the ball-screw electro-mechanical servo system, DDVC-FRVSG faces characteristics of hydraulic-like oil compressibility, temperature effects, etc., thereby proposing a method to solve such problems. The future research will focus on improving the dynamic performance of DDVC-FRVSG with the aim to eliminate the nonlinear phenomena. Consequently, we can improve the dynamic performance, enhancing the accuracy and effectiveness of the compensation for the friction model.
